# Coexistence of *bla*_NDM-1_ and *bla*_IMP-4_ in One Novel Hybrid Plasmid Confers Transferable Carbapenem Resistance in an ST20-K28 *Klebsiella pneumoniae*

**DOI:** 10.3389/fmicb.2022.891807

**Published:** 2022-05-31

**Authors:** Xinmiao Jia, Peiyao Jia, Ying Zhu, Wei Yu, Xue Li, Jingyuan Xi, Xiaoyu Liu, Kang Liao, Yingchun Xu, Bin Cheng, Qiwen Yang

**Affiliations:** ^1^Medical Research Center, State Key Laboratory of Complex Severe and Rare Diseases, Peking Union Medical College Hospital, Chinese Academy of Medical Sciences and Peking Union Medical College, Beijing, China; ^2^Department of Clinical Laboratory, State Key Laboratory of Complex Severe and Rare Diseases, Peking Union Medical College Hospital, Chinese Academy of Medical Sciences and Peking Union Medical College, Beijing, China; ^3^Graduate School, Peking Union Medical College, Chinese Academy of Medical Sciences, Beijing, China; ^4^Department of Clinical Laboratory, Beijing Anzhen Hospital, Capital Medical University, Beijing, China; ^5^Department of Clinical Laboratory Center, Beijing Children’s Hospital, National Center for Children’s Health, Capital Medical University, Beijing, China; ^6^Department of Laboratory Medicine, The First Affiliated Hospital of Sun Yat-sen University, Guangzhou, China; ^7^Department of Clinical Laboratory, Miyun Teaching Hospital, Capital Medical University, Beijing, China

**Keywords:** carbapenemase, NDM, IMP, *Klebsiella pneumoniae*, plasmid

## Abstract

**Objectives:**

We identified a novel hybrid plasmid simultaneously carrying *bla*_NDM-1_ and *bla*_IMP-4_ in an ST20-K28 carbapenem-resistant *Klebsiella pneumoniae* (CRKP) strain AZS099 and reported its detailed genetic and phenotypic characterization.

**Methods:**

Antimicrobial susceptibility was characterized using broth microdilution method. Complete genome characteristics and plasmid detailed analysis were carried out by PacBio Sequel and Illumina sequencing and further bioinformatics analysis. Conjugation assay, S1-PFGE, Southern blot, plasmid stability, and fitness cost were conducted to the phenotypic characterization of this novel hybrid plasmid.

**Results:**

AZS099 was isolated from a blood specimen obtained from a 3-month baby who presented with biliary tract infection. Susceptibility testing showed that AZS099 was resistant to almost all β-lactams examined, including cephalosporins, combinations of β-lactams and β-lactamase inhibitors, carbapenems, and aztreonam. PacBio and Illumina sequencing together with S1-PFGE and Southern blot showed that *bla*_NDM-1_ and *bla*_IMP-4_ were simultaneously located on a 296 kb IncFIB(K)/IncHI1B/IncX3 plasmid (pAZS099-NDM-IMP), which consists of four main parts that came from four different types of plasmids. The region harboring *bla*_IMP-4_ is located in a class 1 integron designated as *In0*, which is located in an *IS6100*-*IS26* transposon-like structure with a total length of ~5 kb. The region harboring *bla*_NDM-1_ is located in the *Tn125* transposon remnant. Conjugation and transformation assay confirmed that the plasmid pAZS099-NDM-IMP has the potential for horizontal transfer and displayed high stability (retention rate > 95%). Furthermore, growth curve assessment confirmed that the presence of pAZS099-NDM-IMP exhibits no growth pressure on bacteria.

**Conclusion:**

Our research reported a hybrid plasmid coharboring *bla*_NDM-1_ and *bla*_IMP-4_ in an ST20-K28 CRKP strain. The emergence of novel hybrid plasmid could threaten the control of antimicrobial resistance and should be closely supervised.

## Highlights

- The first detailed report of an ST20-K28 bloodstream infection carbapenem-resistant *Klebsiella pneumoniae* (CRKP) strain with two carbapenemases (IMP-4 and NDM-1) simultaneously located on one hybrid plasmid.
- The complete genome and detailed structure analysis of this hybrid plasmid co-harboring *bla*_NDM-1_ and *bla*_IMP-4_.
- Complete functional verification experiments, including S1-PFGE, Southern blot, conjugation, plasmid stability, and fitness cost.

## Introduction

*Klebsiella pneumoniae* (*K. pneumoniae*) is a significant pathogen causing serious nosocomial and community-acquired (Jones, 2010; Aiken et al., 2011; Laupland and Church, 2014) infections worldwide. It can cause a broad range of infections (Martin and Bachman, 2018), including pneumonia, urinary tract infection, liver abscess, and bloodstream infection (Navon-Venezia et al., 2017; Huang et al., 2018; Wyres et al., 2020).

In recent years, due to the widespread use of broad-spectrum antimicrobial drugs, *K. pneumoniae* has also shown multiple drug resistance, especially carbapenem resistance, representing a serious threat to human health and a serious challenge for clinicians (Meatherall et al., 2009). Moreover, carbapenem-resistant *K. pneumoniae* (CRKP) has led to severe infections in weak and immunocompromised patients around the world, resulting in prolonged length of hospital stays and increased mortality (Tzouvelekis et al., 2012). The primary type of CRKP is the high-risk clonal complex (CC) 258, including sequence type (ST) 258, ST11, and some closely related STs. Additionally, other clones may also give rise to the international spread of CRKP, and ST20 CRKP was firstly reported in 2020 (Qiao et al., 2020).

The main mechanism of carbapenem resistance in *K. pneumoniae* is the acquisition of carbapenemases (carbapenem hydrolyzing enzymes). The predominant carbapenemase in *K. pneumoniae* is KPC (class A serine β-lactamase), followed by class B metallo β-lactamase NDM and IMP (Wang et al., 2018). Both NDM and IMP can hydrolyze a broad spectrum of β-lactams covered penicillins, cephalosporins, even carbapenems. Not only that, strains containing NDM and IMP are also resistant to ceftazidime/avibactam (Bush, 2013; Bush and Bradford, 2019). Although IMP-producing CRKP and NDM-producing CRKP have been widely identified, CRKP with the coexistence of IMP-4 and NDM-1 has been rarely reported. To our knowledge, only three IMP-4 and NDM-1 co-producing CRKP isolates have been identified (Chen et al., 2015; Liu et al., 2018). The strains belong to ST1043, ST571, and ST273, respectively. In the ST273 strain, the structure of the plasmid was further characterized, and two carbapenemase genes are located on two different plasmids (*bla*_IMP-4_ carried by a self-transmissible IncHI5 plasmid and *bla*_NDM-1_ located on an IncN self-transmissible plasmid). Plasmid-encoded NDM/IMP poses a critical clinical threat because they are carried by mobile genetic elements which can harbor and spread resistance genes to other antimicrobial classes (Bush and Bradford, 2019). However, so far, the detailed genomes of CRKP strains with two enzymes (NDM-1 and IMP-4) simultaneously located on the same plasmid have not been reported. In this study, we identified an ST20 CRKP clinical strain AZS099 simultaneously carrying *bla*_NDM-1_ and *bla*_IMP-4_ genes on one novel hybrid plasmid and firstly report its detailed structure characterization here.

## Materials and Methods

### Bacterial Strains

The strain was isolated on August 21, 2018, from a blood specimen obtained from a 3-month baby who presented with biliary tract infection in Guangzhou, Guangdong, China, and was identified by Vitek MS MALDI-TOF (BioMérieux) system in Peking Union Medical College Hospital. The hemogram indexes are the results of the first examination after admission within 1–3 days of the occurrence of a bloodstream infection. *Escherichia coli* rifampicin-resistant strain EC600 were used in the construction of transconjugants experiments.

### Antimicrobial Susceptibility Testing

We used the broth microdilution method for antimicrobial susceptibility testing as per the Clinical and Laboratory Standards Institute (CLSI) recommendations (CLSI, 2020). MICs were interpreted according to the CLSI M100-S30 guidelines (CLSI, 2020). *Escherichia coli* ATCC 25922, *Pseudomonas aeruginosa* ATCC 27853, and *K. pneumoniae* ATCC700603 were used as quality controls.

### Genomic DNA Extraction, Sequencing, Assembly, Correction, and Annotation

UltraClean^®^ Microbial DNA Isolation Kit (MOBIO Laboratories, Inc.) was used for genomic DNA extraction. Whole-genome sequencing was implemented using the PacBio Sequel platform. A 10 kb SMRTbell library was prepared from sheared genomic DNA (≥5 g) with an additional bead clean-up step before primer annealing (Zhu et al., 2016).

We also re-sequenced this isolate using Illumina sequencing platform in order to correct the polymer errors generated during PacBio sequencing. Paired-end libraries were constructed from 5 μg of isolated genomic DNA using a TruSeq DNA sample prep kit (Illumina Inc., San Diego, California, United States), and sequenced using Illumina with a read length of 2 × 150 bp. A threshold of 0.01 (phred score of 20) was used for raw reads filtration. Genome assembly was conducted using Unicycler (Wick et al., 2017) from short and long sequencing reads.

Genome sequences were initially annotated with the rapid prokaryotic genome annotation software Prokka (Seemann, 2014) and further annotated by BLAST searches against the RefSeq and UniProtKB/Swiss-Prot databases. Pairwise sequence comparisons were also performed using BLAST. All mobile elements were identified using ExPASy (Artimo et al., 2012), ISFinder (Siguier et al., 2006), the Transposon Registry (Tansirichaiya et al., 2019), INTEGRALL (Moura et al., 2009), and Integron Finder.^1^ Comparisons of plasmid structures were conducted to analyze the sequence homology. BRIG software was used for the generation of plasmid circular structure maps.

### Sequence Type, Serotype, Virulence, and Antimicrobial Resistance Genes Analysis

STs were determined using SRST2 (Inouye et al., 2014) based on the Illumina reads. Serotype was analyzed using SerotypeFinder 2.0 (Joensen et al., 2015) based on the assembled contigs. Virulence genes were downloaded from Virulence Factor Database (VFDB). FASTA sequences of the downloaded virulence genes were used for searching corresponding genes by BLAST with coverage of 50% and identity of 90%. Antimicrobial resistance genes were analyzed through ResFinder 4.0 based on the whole genomic sequences.

### Plasmid Conjugation Assay

The transferability of pAZS099-NDM-IMP among isolates was determined using AZS099 as donor and *E. coli* EC600 as the recipient by conjugation assay. The procedure of conjugation was performed according to the protocol previously described (Yang, 2015). 100 μl overnight culture of clinical isolate (AZS099) in Luria–Bertani (LB) broth containing meropenem (2 μg/ml) and 200 μl overnight culture of recipient EC600 in LB broth containing rifampicin (100 μg/ml) were mixed into 5-ml antibiotic-free LB broth and were incubated at 37°C for 18 h. Then, the conjugant mixtures were placed on selective LB agar containing 100 μg/ml rifampin and 1 μg/ml meropenem. PCR analysis and agarose gel electrophoresis were performed to confirm the effects of transconjugation.

### S1-PFGE, Southern Blot, and PCR Amplification and Sanger Sequencing

To further characterize the plasmid pAZS099-NDM-IMP in AZS099 and the transconjugant EC600-pAZS099-NDM-IMP, S1 nuclease digestion analyzed with PFGE was performed using a CHEF Mapper XA apparatus (Bio-Rad Laboratories, Hercules, CA). Briefly, the adjusted bacterial suspension mixed in a 1% Seakem Golden Agarose and 1% Sodium dodecyl sulfate (SDS) was digested with proteinase K for 2 h at 56°C and then further treated with S1 nuclease. XbaI-digested *Salmonella enterica* serovar Braenderup H9812 as a reference molecular weight marker. The electrophoresis conditions used in this study were as follows: initial switch time, 3 s; a final switch time, 36 s; gradient, 6 V/cm; angle, 120; temperature, 14°C; and time, 18 h. After the electrophoresis was complete, the gel was stained with Ultra GelRed and visualized with the ImageQuant LAS 500 (GE LifeScience, Pittsburgh, United States) or transferred to nylon membrane (GE LifeScience, Pittsburgh, United States), followed by hybridization with digoxigenin-labelled probes specific to NDM-1 or IMP-4. Probe labelling and signal detection were done by DIG DNA Labeling and Detection Kit (Roche Diagnostics, GmbH, Germany).

The plasmid pAZS099-NDM-IMP separated by S1 nuclease treatment and pulsed-field gel electrophoresis (PFGE) was then recovered and used as the templates for PCR amplification and Sanger sequencing to identify target genes *bla*_IMP_ (primer sequence 5′-3′: Forward GTAGCATTGCTACCGCAGCAG; Reverse TCGTTTAACCCTTTAACCGCC) and *bla*_NDM_ (primer sequence 5′-3′: Forward TGCCCAATATTATGCACCCG; Reverse CCCAACGGTGATATTGTCACTG) in AZS099 and the transconjugant EC600-pAZS099-NDM-IMP.

### Plasmid Stability Testing

Plasmid stability was assessed upon several successive subculture of plasmid-carrier AZS099 strain for 10 days with 1:1000 dilution on antibiotic-free LB broth at 37°C in a shaking bath (180 rpm). The 10th day’s culture was serially diluted and plated onto LB plates with or without meropenem (1 mg/L). The retention rate of plasmid was calculated by dividing the number of colonies on LB plates supplemented with meropenem by the number of colonies on LB plates (Gao et al., 2020). 50 colonies on the antibiotic-free LB plates were randomly selected and subjected to PCR validation of *bla*_IMP_ (primer sequence 5′-3′: Forward TGGTTTGTGGAACGTGGCTA; Reverse GAGTGATGCGTCTCCAGCTT) and *bla*_NDM_ (primer sequence 5′-3′: Forward CTCGCATTTGCGGGGTTTTT; Reverse ACGCCTCTGTCACATCGAAA) genes.

### Fitness Cost Assessment

Growth curve was used to assess the fitness impact of plasmid carriage under noncompetitive conditions as Wang et al. described previously (Wang et al., 2018). The recipients and transconjugants carrying pAZS099-NDM-IMP plasmid from AZS099 isolate were cultured overnight in LB broth without or with 2 mg/L meropenem at 37°C, respectively. Bacterial suspensions were diluted 1:1000 in LB medium (approximately 10^6^ CFU/ml) and grown at 37°C in triplicate for 36 h by Epoch™ 2 Microplate Spectrophotometer from BioTek Instruments. The OD600 of each culture was measured every 30 min, and the plates were shaken for 15 s before measuring. GraphPad Prism version 8 (GraphPad Software, Inc., United States) was used to estimate the growth curves. Statistical significance was determined for an overall error at 0.05 level (95% confidence interval) using one-way ANOVA, followed by Tukey tests.

## Results

### Clinical Characteristics of Patients With AZS099

The *Klebsiella pneumoniae* strain AZS099 was isolated from a blood specimen obtained from a 3-month baby who presented with biliary tract infection. The patient was hospitalized with cholangitis after biliary atresia and had a fever >39°C lasting for more than 3 days. The total white blood cells (WBC), neutrophil percent (NEU), C-reactive protein (CRP), and procalcitonin (PCT) were 12.85*10^9^/L, 22.7%, 161.71 mg/L, and 1.52 ng/ml, respectively. In the duration of hospitalization, the patient was treated with meropenem (0.15 g, q 8 h, 5 days). Surgical intervention was involved during the treatment, and finally, the patient was improved and discharged.

The results of susceptibility testing showed that the clinical isolate AZS099 was resistant to almost all β-lactams examined, including cephalosporins (cefoxitin, ceftriaxone, ceftazidime, cefepime), carbapenems (imipenem, meropenem, ertapenem), combinations of β-lactams and β-lactamase inhibitors (imipenem/relebactam, piperacillin/tazobactam, ceftazidime/avibactam, ceftolozane/tazobactam), and aztreonam. The MIC of imipenem, meropenem, and ertapenem is >16 μg/ml, >16 μg/ml, and > 4 μg/ml, respectively (Table 1). The strain was susceptible to amikacin, levofloxacin, and colistin (Table 1).

**Table 1:** The minimum inhibitory concentration (MIC) of the clinical strain AZS099 and EC600 with/without plasmid AZS099-NDM-IMP.

Antimicrobial agents^a^	AZS099	EC600	EC600 + pAZS099-NDM-IMP
Colistin	≤0.5	≤0.5	≤0.5
Ertapenem	>4	≤0.06	>4
Cefoxitin	>16	≤2	>16
Imipenem	>16	0.25	16
Ceftriaxone	>8	≤0.5	>8
Piperacillin-tazobactam	>64/4	≤2/4	>64/4
Ceftazidime	>16	≤0.5	>16
Ceftazidime/avibactam	>16/4	0.25/4	>16/4
Meropenem	>16	≤0.06	>16
Ceftolozane/tazobactam	>16/4	0.5/4	>16/4
Cefepime	>16	≤0.5	>16
Amikacin	≤4	≤4	≤4
Levofloxacin	≤0.25	≤0.25	≤0.25
Aztreonam	>8	≤0.5	>8
Imipenem/relebactam	>16/4	0.25/4	16/4

a The MIC (*μ*g/ml) for the antibiotics chosen was determined for each bacterial isolate.

### Genomic Characteristics and Antimicrobial-Resistance Genotype Analysis

**Figure 1:**
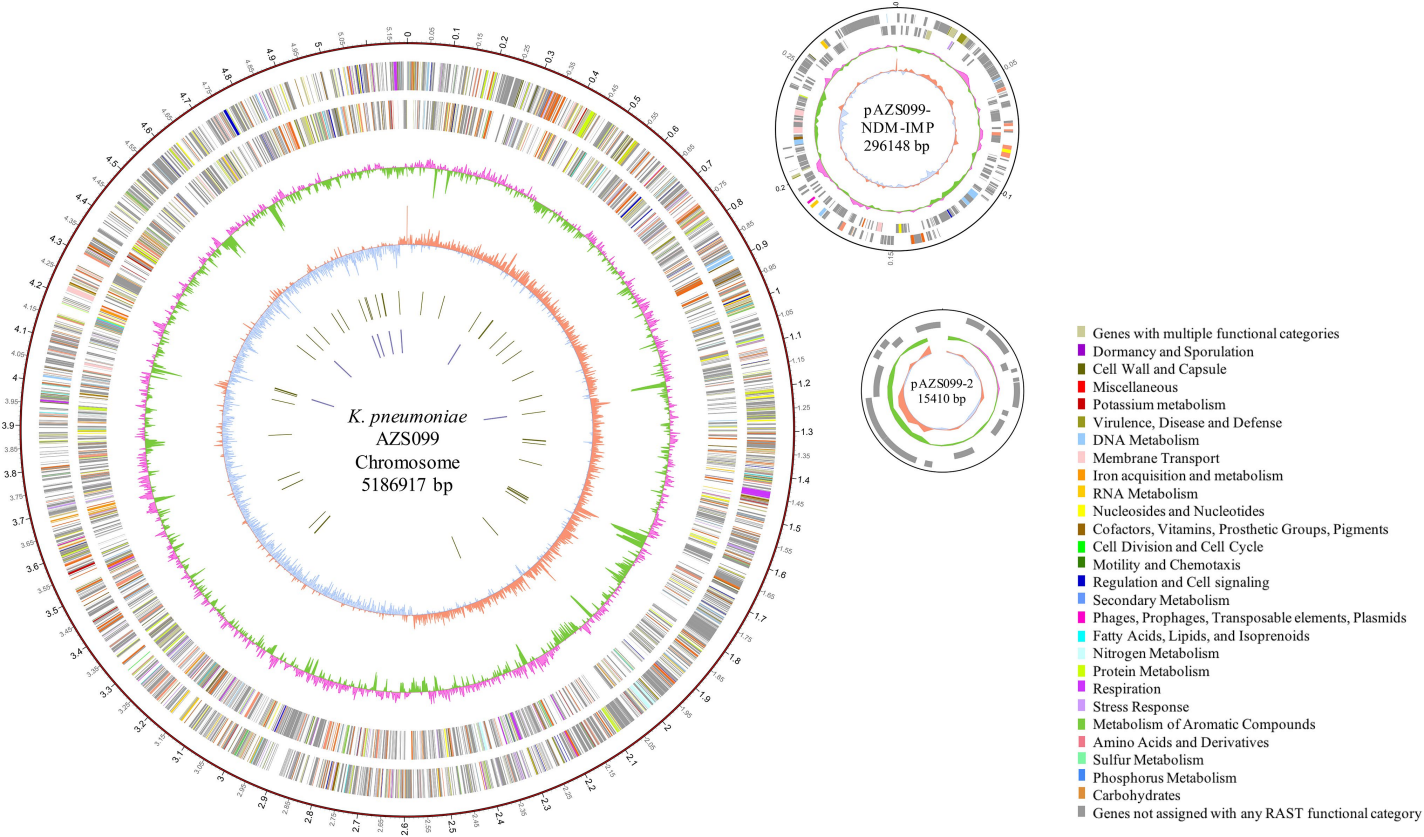
Circular representation of AZS099 genome (chromosome and two plasmids). Circles are shown as follows (outside to inside): (1) A physical map scaled in megabases (Mb) from base 1 (the start of the putative replication origin); (2) coding sequences transcribed in a clockwise direction; (3) coding sequences transcribed in a counter-clockwise direction; (4) G + C content; (5) GC skew; (6) tRNA genes; and (7) rRNA genes. Genes displayed in (2) and (3) are color-coded according to different functional categories shown on the right.

To reveal the genetic basis of the multidrug-resistant (MDR) phenotype, we obtained the complete genome of the clinical isolate AZS099 including a 5.2-Mb chromosome, a 296-kb plasmid (pAZS099-NDM-IMP), and a 15-kb plasmid (pAZS099-2; Table 2 and Figure 1). Bioinformatics analysis provided the general information of the chromosome genome, including GC% content (57.63%), predicted protein-coding genes (4,998), average gene length (922 bp), and coding region (88.82%). The two plasmids possess a shorter average gene length, lower ratio of coding regions, and lower GC% content. Sequence type (ST) and serotype analysis showed that AZS099 belongs to ST20 and K28.

**Table 2:** The genomic characteristics and antimicrobial resistance genotype analysis of the sequences of chromosomes and plasmids.

Name*	Genome size (bp)	GC content	Coding genes	Average gene size (bp)	Coding region (bp)	tRNA	rRNA	Drug resistance genes
AZS099-chr	5,186,917	57.63%	4,998	922	4,606,986 (88.82%)	86	25	*fosA*, *oqxA*, *oqxB*, *bla*_SHV-12_
pAZS099-NDM-IMP	296,148	50.91%	331	731	241,965 (81.70%)	0	0	*bla*_NDM-1_, *bla*_IMP-4_, *bla*_SHV-12_,
								*aph(3″)-Ib*, *aph(6)-Id*, *sul2*, *ble*_MBL_
pAZS099-2	15,410	45.55%	17	620	10,553 (68.48%)	0	0	/

* Sequences of chromosomes end with the word “chr;” sequences of plasmids begin with the word “p.”

Virulence genes (Table 2) analysis showed that the clinical isolate AZS099 only contains nine virulence genes, which are all located on the 5.2-Mb chromosome, including ferric aerobactin receptor protein (*iutA*), and type III fimbria proteins (*mrkABCDFHIJ*) that are related to cell adhesion. No virulence genes were discovered on two plasmids. Eleven antimicrobial-resistance genes were found in AZS099 including four antimicrobial-resistance genes on the chromosome: *fosA*, glutathione transferase encoding gene; *oqxA* and *oqxB*, multidrug efflux pump protein encoding genes; *bla*_SHV-12_, β-lactamase encoding gene. Seven antimicrobial-resistance genes on the 296-kb plasmid (pAZS099-NDM-IMP): two aminoglycoside-resistance genes [*aph(6)-Id* and *aph(3″)-Ib*], one sulfonamide resistance gene (*sul2*), one bleomycin resistance gene (*ble*_MBL_), one β-lactamase (*bla*_SHV-12_), and importantly two carbapenemase resistance gene *bla*_NDM-1_ and *bla*_IMP-4_ (Table 2). These antimicrobial-resistance genes, especially *bla*_NDM-1_ and *bla*_IMP-4_, are responsible for the MDR phenotype, including most β-lactam drugs, which is in accordance with antimicrobial susceptibility testing. No antimicrobial resistance genes were found on the 15-kb plasmid (pAZS099-2).

### Characterization of the Novel Hybrid Plasmid pAZS099-NDM-IMP Co-harboring *bla*_NDM-1_ and *bla*_IMP-4_

**Figure 2:**
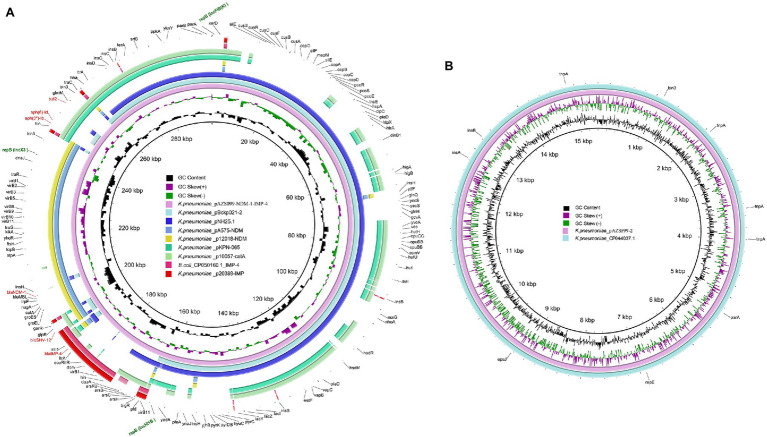
Structural comparison of pAZS099-NDM-IMP and pAZS099-2. **(A)** Structural comparison of pAZS099-NDM-IMP and eight plasmids pNH25.1 (CP024875.1), pBckp021-2 (CP050836.1), p20389-IMP (MH909337.1), CP050160.1_IMP-4, pA575-NDM (MH917283.1), p12018-NDM (MH909343.1), pKPN-065 (CP015026.1), and p10057-catA (MN423364.1). **(B)** Structural comparison of pAZS099-2 and plasmid CP044037.1. Alignments of resemble plasmids are shown as concentric rings. The outermost shows the main coding genes of pAZS099-NDM-IMP. Antimicrobial-resistance genes are highlighted in red.

Antimicrobial-resistance genes analysis showed both *bla*_NDM-1_ and *bla*_IMP-4_ are located on the 296-kb plasmid (pAZS099-NDM-IMP), which was further confirmed by S1-PFGE, Southern blot, and PCR amplification (Supplementary Figures S1–S3). The complete nucleotide sequence of the plasmid pAZS099-NDM-IMP was 296,148 bp in length, constituting a circular DNA with an average G + C content of 50.91% and 331 open reading frames (Table 2). This plasmid pAZS099-NDM-IMP contained three types of plasmid replication initiation genes: IncFIB(K), IncHI1B and IncX3. We performed a full-plasmid BLAST comparative analysis in NCBI, but no plasmids with a similar structure as pAZS099-NDM-IMP were found. Further analysis showed that the sequence of pAZS099-NDM-IMP consists of four main parts, which were came from four different types of plasmids (Figure 2A): the main backbone region (1 bp - ~175,000 bp) shared >99% identity with pNH25.1 (CP024875.1) and pBckp021-2 (CP050836.1); the *bla*_IMP-4_ region (~175,000 bp– ~190,000 bp) shared >99% identity with p20389-IMP (MH909337.1) and CP050160.1_IMP-4 of *E. coli*; the *bla*_NDM-1_ region (~190,000 bp - ~245,000 bp) shared >99% identity with pA575-NDM (MH917283.1) and p12018-NDM (MH909343.1); the aminoglycoside resistance region (~245,000–296,148 bp) shared >99% identity with pKPN-065 (CP015026.1) and p10057-catA (MN423364.1). These four parts make up the new hybrid plasmid.

**Figure 3:**
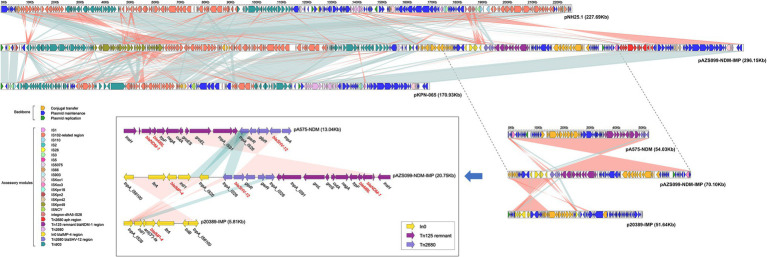
Structure of plasmid pAZS099-NDM-IMP coharboring *bla*_NDM-1_ and *bla*_IMP-4_ and related transposon and integron. Linear comparison of plasmid pAZS099-NDM-IMP with four most similar plasmids (pNH25.1, pKPN-065, pA575-NDM, and p20389-IMP) and structure comparison of transposon *Tn125* remnant containing *bla*_NDM-1_ and integron *In0* containing *bla*_IMP-4_. Genes are denoted by arrows. Genes, mobile elements, and other features are colored based on functional classification. Shading denotes the regions with high homology (95% nucleotide identity).

A linear genomic comparison and mobile genetic element (MGE) annotation were further conducted among pAZS099-NDM-IMP, pNH25.1, pKPN-065, pA575-NDM (*bla*_NDM-1_-carrying plasmid), and p20389-IMP (*bla*_IMP-4_-carrying plasmid; Figure 3) since they share the high identity and both belong to *K. pneumoniae*. Although the region 1 bp– ~175,000 bp and ~ 245,000–296,148 bp shared high identity with pNH25.1 and pKPN-065, many inversion regions were identified. *bla*_NDM-1_ and *bla*_IMP-4_ are located in a 70 kb region, which also contains a β-lactamase *bla*_SHV-12_. There is an inversion of the accessory modules containing *bla*_IMP-4_ compared with the *bla*_IMP-4_ region of p20389-IMP, and an inversion of the accessory modules containing *bla*_NDM-1_ was also identified compared with the *bla*_NDM-1_ region of pA575-NDM. Additionally, this 70 kb region covered almost 100% of the IncX3-type plasmid pA575-NDM, and the *bla*_IMP-4_ region was inserted into downstream of this region (Figures 2A, 3). Detail MGE annotation showed that (Figure 3) the region harboring *bla*_IMP-4_ is located in a class 1 integron designated as *In0*, which is carried by an *IS6100-IS26* transposon-like structure with a total length of ~5 kb. This integron also contains the retron-type RNA-directed DNA polymerase *ltrA*. Compared to the integron *In0* in p20389-IMP, *In0* in this hybrid plasmid pAZS099-NDM-IMP lost *ant(3″)-Ia* and *tniB*. The region harboring *bla*_NDM-1_ is located in the *Tn125* transposon remnant. This region also contained a bleomycin resistance protein (*ble*_MBL_), *trpF*, *nagA*, *cutA*, *groS*, and *groL*. Additionally, *Tn2680* (with two *IS26* elements at terminal regions in opposite directions), carrying β-lactamase *bla*_SHV-12_, is located between the *Tn125* remnant region containing *bla*_NDM-1_ and integron *In0* containing *bla*_IMP-4_. Integron *In0*, *Tn2680*, and *Tn125* remnant region lay adjacent to one another and formed a continuous 20 kb multidrug-resistant region that confers resistance to almost all β-lactams examined, containing cephalosporins, combinations of β-lactams and β-lactamase inhibitors, carbapenems, aztreonam, and ampicillin.

We also performed a full-plasmid BLAST comparative analysis of pAZS099-2 in NCBI, and it exhibited 99% identity with a plasmid from *K. pneumoniae* strain FDAARGOS_630 (CP044037.1; Figure 2B). This plasmid (pAZS099-2) is IncFIA(HI1) type, 15,410 bp in length, constituting a circular DNA with an average G + C content of 45.55% and 17 open reading frames. No virulence and antimicrobial-resistance genes were found.

### Transferability, Stability, and Fitness Cost of the Resistance Plasmid (pAZS099-NDM-IMP)

To evaluate the transferability of the resistance plasmid carrying *bla*_NDM-1_ and *bla*_IMP-4_, conjugation assays were performed by co-culturing AZS099 with *E. coli* EC600. S1-PFGE confirmed the size of the plasmid, which was consistent with the donor AZS099, in the transconjugant (Supplementary Figure S1A). The following Southern blot and PCR results confirmed the coexistence of *bla*_NDM-1_ and *bla*_IMP-4_ on the plasmid pAZS099-NDM-IMP (Supplementary Figures S1B,C, S2, S3). These results indicated that the plasmid pAZS099-NDM-IMP harboring *bla*_NDM-1_ and *bla*_IMP-4_ in the clinical strain AZS099 has the potential for horizontal transfer. Moreover, the two carbapenem resistance genes *bla*_NDM-1_ and *bla*_IMP-4_ were also transferred along with the plasmid. As shown in Table 1, the susceptibility results of the transconjugant were consistent with the donor (clinical isolate AZS099): resistant to cephalosporins, carbapenems, combinations of β-lactams and β-lactamase inhibitors. And the MIC range of meropenem for NDM-IMP-bearing transconjugants was >16 μg/ml.

The stability of plasmid pAZS099-NDM-IMP was also evaluated during serial passage in the laboratory last for 10 days. It displayed high stability, as the retention rates were still over 95% at the end of the experiment for both *bla*_NDM-1_ and *bla*_IMP-4_, which were confirmed by PCR.

**Figure 4:**
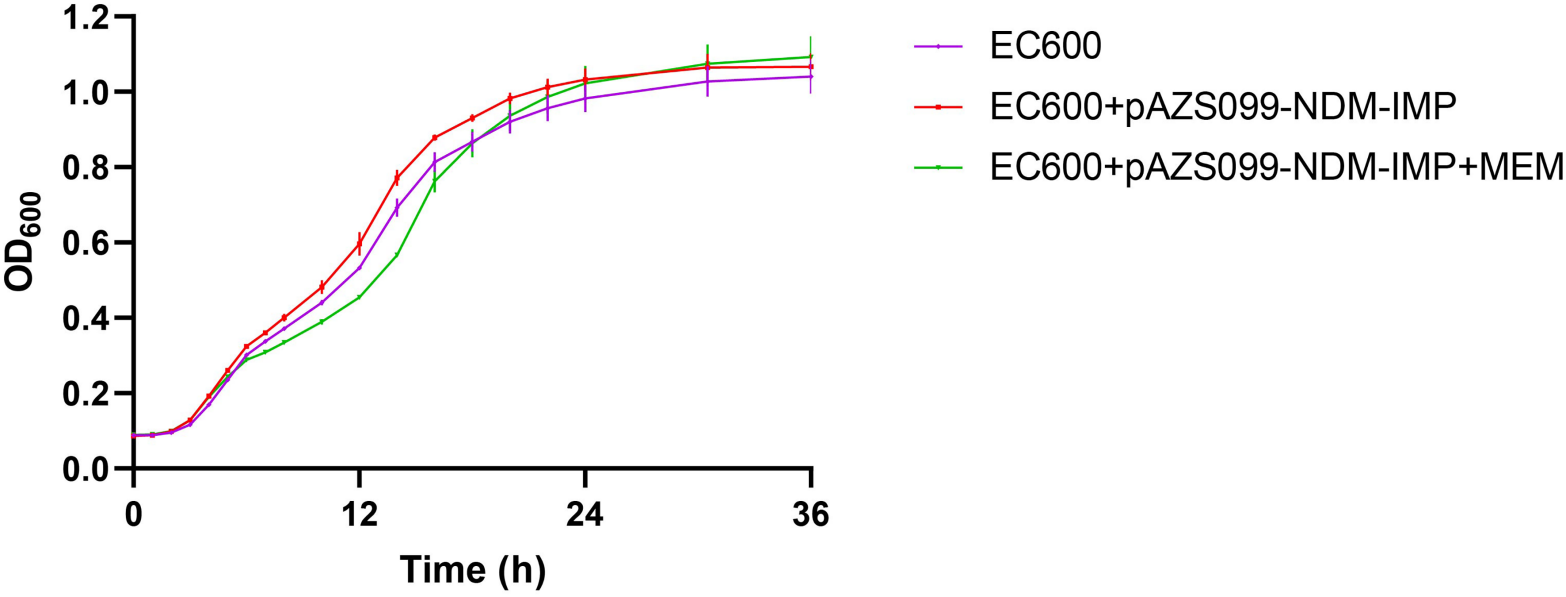
Growth curve comparison. Growth curves of recipient strain *E. coli* EC600 and the transconjugants harboring *bla*_NDM-1_ and *bla*_IMP-4_ plasmid pAZS099-NDM-IMP with and without meropenem (value of *p* >0.05). EC600 means the recipient strain *E. coli* EC600. EC600 + pAZS099-NDM-IMP means the transconjugant harboring *bla*_NDM-1_ and *bla*_IMP-4_ plasmid pAZS099-NDM-IMP in antibiotic-free LB broth. EC600 + pAZS099-NDM-IMP+MEM means the transconjugant harboring *bla*_NDM-1_ and *bla*_IMP-4_ plasmid pAZS099-NDM-IMP in LB agar plates containing meropenem.

Further, the effect of acquiring *bla*_NDM-1_ and *bla*_IMP-4_ plasmid on biological fitness cost was evaluated. Of interest, no significant differences in the growth rate were observed among the recipient strain EC600 and the transconjugant harboring *bla*_NDM-1_ and *bla*_IMP-4_ plasmid pAZS099-NDM-IMP (*p* > 0.05; Figure 4). Therefore, the antibiotic resistance mediated by pAZS099-NDM-IMP did not increase the growth fitness cost of the resistant strains compared to their susceptible counterparts.

## Discussion

In this study, we identified an ST20-K28 bloodstream infection CRKP carrying a novel hybrid plasmid coharboring *bla*_NDM-1_ and *bla*_IMP-4_ and characterized the detailed structure of this hybrid plasmid. Moreover, we assessed the transferability, stability, and impact on the strain survival of the plasmid.

The continuous and often inappropriate use of β-lactam drugs has led to the emergence and global spread of extended-spectrum β-lactamases (ESBLs) and, more recently, carbapenemases, which limits the use of all β-lactam agents. According to previous epidemiological studies, the most commonly identified carbapenemases in *K. pneumoniae* is KPC-2 (Wang et al., 2018; Yang et al., 2020). And *K. pneumoniae* strains with *bla*_KPC-2_ have been widely disseminated in several regions, including Europe, South and North America, and Asia (Nordmann et al., 2009). Additionally, the emergence of metallo-β-lactamases (MBLs) NDM-1 and IMP-4 has also become an established major public health, posing new challenges to the treatment of infectious diseases. MBLs are of great concern due to their carbapenemase activity, their rapid spread in major human opportunistic pathogens, while no clinically useful inhibitor is available yet (Carcione et al., 2021; Legru et al., 2021). Avibactam is one of the most important drugs and inhibits β-lactamases of class A, including KPC, class C, and certain class D β-lactamases, mainly OXA-48. Unfortunately, it is not active against MBL enzymes (β-lactamases of class B) (Tooke et al., 2019; Carcione et al., 2021). Researchers to develop MBL inhibitors have been focused on compounds that bind and/or chelate zinc ions at the active site, but the high heterogeneity of these enzymes poses a serious problem. Currently, no drugs are close to the clinic (Tooke et al., 2019; Gonzalez-Bello et al., 2020; Carcione et al., 2021; Davies and Everett, 2021).

At present, IMP-producing CRKP and NDM-producing CRKP have been widely identified. However, CRKP with the coexistence of IMP-4 and NDM-1 has been rarely reported. To our knowledge, only three IMP-4 and NDM-1 co-producing CRKP isolates have been identified in Southeast Asia (Chen et al., 2015; Liu et al., 2018). In 2015, Chen et al. reported the coexistence of *bla*_NDM-1_ and *bla*_IMP-4_ in two *K. pneumoniae* isolates (ST1043 and ST571). However, the sequence features of plasmids carrying these two genes were not characterized (Chen et al., 2015). In 2018, Liu et al. reported a ST273 CRKP carrying *bla*_NDM-1_ and *bla*_IMP-4_. The two carbapenemase genes are located on two different plasmids (Liu et al., 2018). Except for *K. pneumoniae*, Zhang et al. reported a KPC-2-, NDM-1-, and IMP-4-producing *K. michiganensis* isolate recently, and *bla*_KPC-2_, *bla*_NDM-1_, and *bla*_IMP-4_ were carried on different conjugative plasmids (Zhang et al., 2022). Moreover, Li et al. described a hybrid plasmid co-harboring *bla*_NDM-1_ and *bla*_IMP-4_ from *K. michiganensis* (Li et al., 2021). However, no similar plasmids were reported in *K. pneumoniae*. Our study, for the first time, reported a CRKP strain with the two enzymes (IMP-4 and NDM-1) simultaneously located on one hybrid plasmid and characterized the detailed structure of this hybrid plasmid (pAZS099-NDM-IMP). Additionally, the MIC of meropenem, imipenem, and ertapenem for AZS099 is >16 μg/ml, > 16 μg/ml, and > 4 μg/ml, respectively. We noticed that the MIC of both meropenem and imipenem for most reported NDM-1 harboring *K. pneumoniae* strains is 8 μg/ml (Zhang et al., 2015, 2018). And the MIC of ertapenem for IMP-4 harboring *K. pneumoniae* strains is >4 μg/ml, while it is <8 μg/ml of meropenem and imipenem (Limbago et al., 2011; Lai et al., 2017). This shows that there is an increase in MIC value of the strain AZS099 coharboring *bla*_NDM-1_ and *bla*_IMP-4_. Although the patient was eventually cured and discharged, which might be due to timely surgical drainage, followed by appropriate ICU care and early goal directed therapy, this kind of strain should also be paid more attention.

Moreover, the spread of plasmid-mediated carbapenem-resistant clinical isolates is a severe threat to global health. The two enzymes (IMP-4 and NDM-1) are located on the same plasmid, which makes it easier for the two enzymes to spread at the same time. Our result also implied that the hybrid plasmid co-harboring *bla*_NDM-1_ and *bla*_IMP-4_ is stable and has the potential for horizontal transfer. In addition, this hybrid plasmid did not increase the growth fitness cost of the clinical strain, which highlights the importance of controlling the spread of NDM-1-IMP-4-CRKPs. Detailed plasmid structure analysis showed that the plasmid replicon type of pAZS099-NDM-IMP was IncFIB(K)/IncHI1B/IncX3. *bla*_NDM-1_ came from the IncX3-type plasmid similar to pA575-NDM and *bla*_IMP-4_ was integrated into this region. The IncX3-type plasmid has been found to be widely distributed in various species in different regions of China and the United Arab Emirates, and may contribute to the spread of *bla*_NDM-1_ (Qu et al., 2015). In our strain, *bla*_NDM-1_ is located in the *Tn125* transposon remnant without *ISAba125* fragment. We speculated that *bla*_NDM-1_ might become a relatively fixed component on the plasmid and disseminated together with IncX3 plasmid (Ho et al., 2012). In this hybrid plasmid, the *bla*_IMP-4_ gene in class 1 integron *In0* flanked by *IS26* and *IS6100* on both sides. Class 1 integrons are responsible for the dissemination of the *bla*_IMP_ gene (Huang et al., 2021). Besides, the insertion sequence also plays important roles in the transmission of resistant genes. Previous studies on *bla*_IMP-4_-carrying plasmids showed that *IS26* might play important roles in disseminating *bla*_IMP-4_ in different plasmids (Liu et al., 2021). Moreover, the region of *bla*_NDM-1_, *bla*_SHV-12_ and *bla*_IMP-4_ lay adjacent to one another and formed a continuous 20 kb multidrug-resistant region. We further compared pAZS099-NDM-IMP (296,148 bp) with the reported hybrid plasmid (pKOC7525_1, 397,447 bp) co-harboring *bla*_NDM-1_ and *bla*_IMP-4_ in *K. michiganensis* (Li et al., 2021). The result showed that the plasmid sequences and structure of the two strains were different from each other, and pKOX7525_1 only covered 25.67% of pAZS099-NDM-IMP (Supplementary Figures S4, S5), which indicated the different sources of these two *bla*_NDM-1_ and *bla*_IMP-4_ co-harboring plasmids. Moreover, *bla*_NDM-1_ and *bla*_IMP-4_ are ~100 kb apart from each other on the plasmid (pKOC7525_1) of *K. michiganensis*, while it is only ~20 kb on pAZS099-NDM-IMP, which might greatly increase the risk of simultaneous transfers of this multidrug-resistant region in *K. pneumoniae*. We also compare pAZS099-NDM-IMP with the plasmids containing NDM-1 (pK210011_NDM) and IMP-4 (pK210011_IMP) in *K. michiganensis* isolate K210011 reported by Zhang et al. recently (Zhang et al., 2022). In *K. michiganensis* isolate K210011, *bla*_NDM-1_, and *bla*_IMP-4_ were carried by different conjugative plasmids, and pK210011_NDM covered 31.83%, pK210011_IMP covered 17.87% of pAZS099-NDM-IMP, respectively (Supplementary Figures S4, S5). These results further illustrate the novelty of the plasmid pAZS099-NDM-IMP.

Our research reported a hybrid plasmid coharboring *bla*_NDM-1_ and *bla*_IMP-4_ in an ST20-K28 CRKP strain. The emergence of novel hybrid plasmid may contribute to the further dissemination of carbapenem-resistant genes and pose new challenges to the treatment of infectious diseases. Surveillance of carbapenemases especially MBLs, in *K. pneumoniae* is urgently needed to control and prevent the spread of these resistance determinants in China.

## Data Availability Statement

The complete genome sequences of strain AZS099 were deposited at GenBank (CP086761–CP086763).

## Author Contributions

XJ and QY conceived and designed the study. XJ, PJ, YZ, WY, XLi, JX, and XLiu performed the experiments and analyzed the data. KL contributed the isolate. XJ, PJ, YX, BC, and QY prepared the manuscript. All authors approved the final manuscript. All authors contributed to the article and approved the submitted version.

## Funding

This work was supported by the National Natural Science Foundation of China (82072318 and 32000463), National Key Research and Development Program of China (2021YFC2301002 and 2018YFE0101800), Beijing Key Clinical Specialty for Laboratory Medicine — Excellent Project (No. ZK201000).

## Conflict of Interest

The authors declare that the research was conducted in the absence of any commercial or financial relationships that could be construed as a potential conflict of interest.

## Publisher’s Note

All claims expressed in this article are solely those of the authors and do not necessarily represent those of their affiliated organizations, or those of the publisher, the editors and the reviewers. Any product that may be evaluated in this article, or claim that may be made by its manufacturer, is not guaranteed or endorsed by the publisher.

## Supplementary Material

The Supplementary Material for this article can be found online at: https://www.frontiersin.org/articles/10.3389/fmicb.2022.891807/full#supplementary-material

## Footnotes

^1^ https://github.com/gem-pasteur/Integron_Finder
