# Supplementary material for: Coexistence of blaNDM-1 and blaIMP-4 in One Novel Hybrid Plasmid Confers Transferable Carbapenem Resistance in an ST20-K28 Klebsiella pneumoniae
Source: Front Microbiol. 2022 May 31;13:891807. doi: 10.3389/fmicb.2022.891807 (PMC9194606; doi:10.3389/fmicb.2022.891807)
Supplement: Supplementary file 1 [file Data_Sheet_1.DOCX]

**Figure S1**


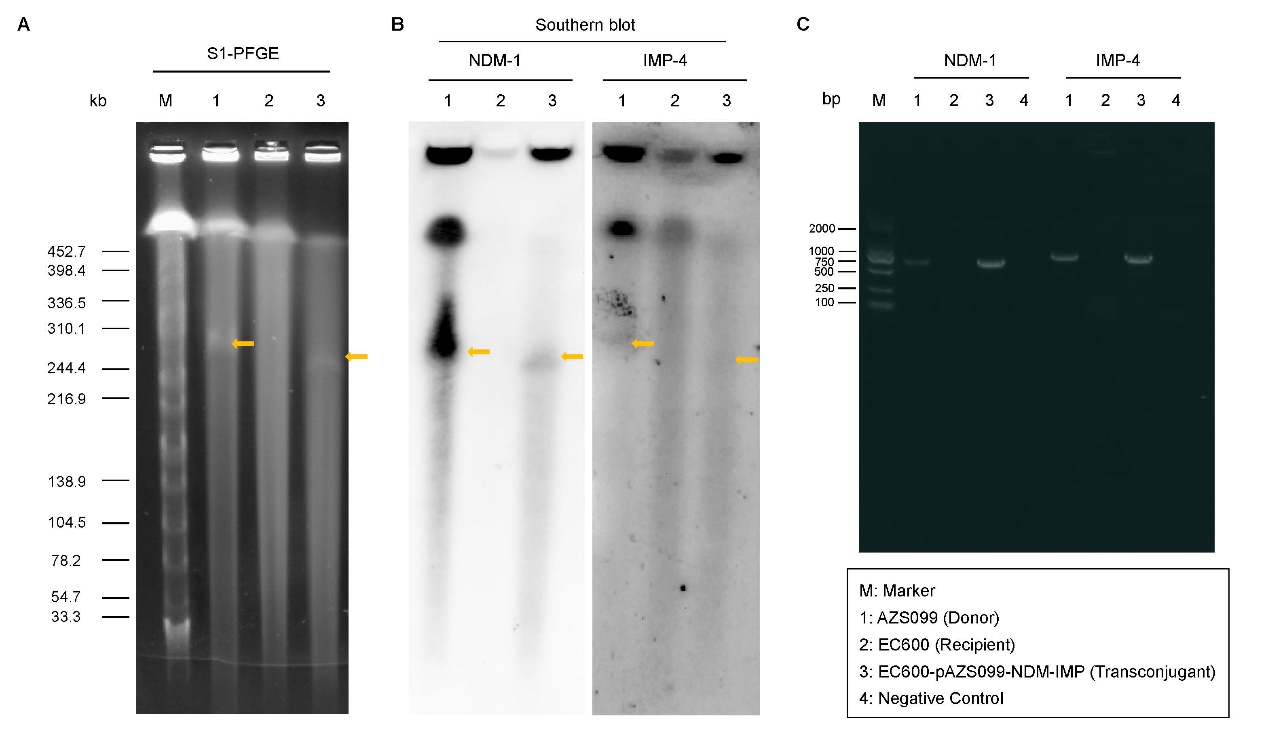


**Figure S1. Confirmation of the co-existence of NDM-1 and IMP-4 on the plasmid pAZS099-NDM-IMP and the transconjugant.** (A) S1-PFGE of *K. pneumoniae* AZS099, the recipient strain *E. coli* EC600 and the transconjugant EC600-pAZS099-NDM-IMP harboring *bla*_NDM-1_ and *bla*_IMP-4_ plasmid pAZS099-NDM-IMP. Yellow arrows denote the position of the hybrid plasmid pAZS099-NDM-IMP. The plasmid length of pAZS099-NDM-IMP in S1-PFGE is consistent with genome assembly result. (B) Confirmation of the co-existence of NDM-1 and IMP-4 on the plasmid pAZS099-NDM-IMP and the transconjugant by Southern blot. Yellow arrows denote the position of NDM-1 and IMP-4 in pAZS099-NDM-IMP from AZS099 and the transconjugant EC600-pAZS099-NDM-IMP, which is consistent with S1-PFGE. (C) Confirmation of the co-existence of NDM-1 and IMP-4 on the plasmid pAZS099-NDM-IMP and the transconjugant by PCR amplifications. The plasmid pAZS099-NDM-IMP separated by S1 nuclease treatment and PFGE was then recovered and used as the templates for PCR amplification.

**Figure S2**

**
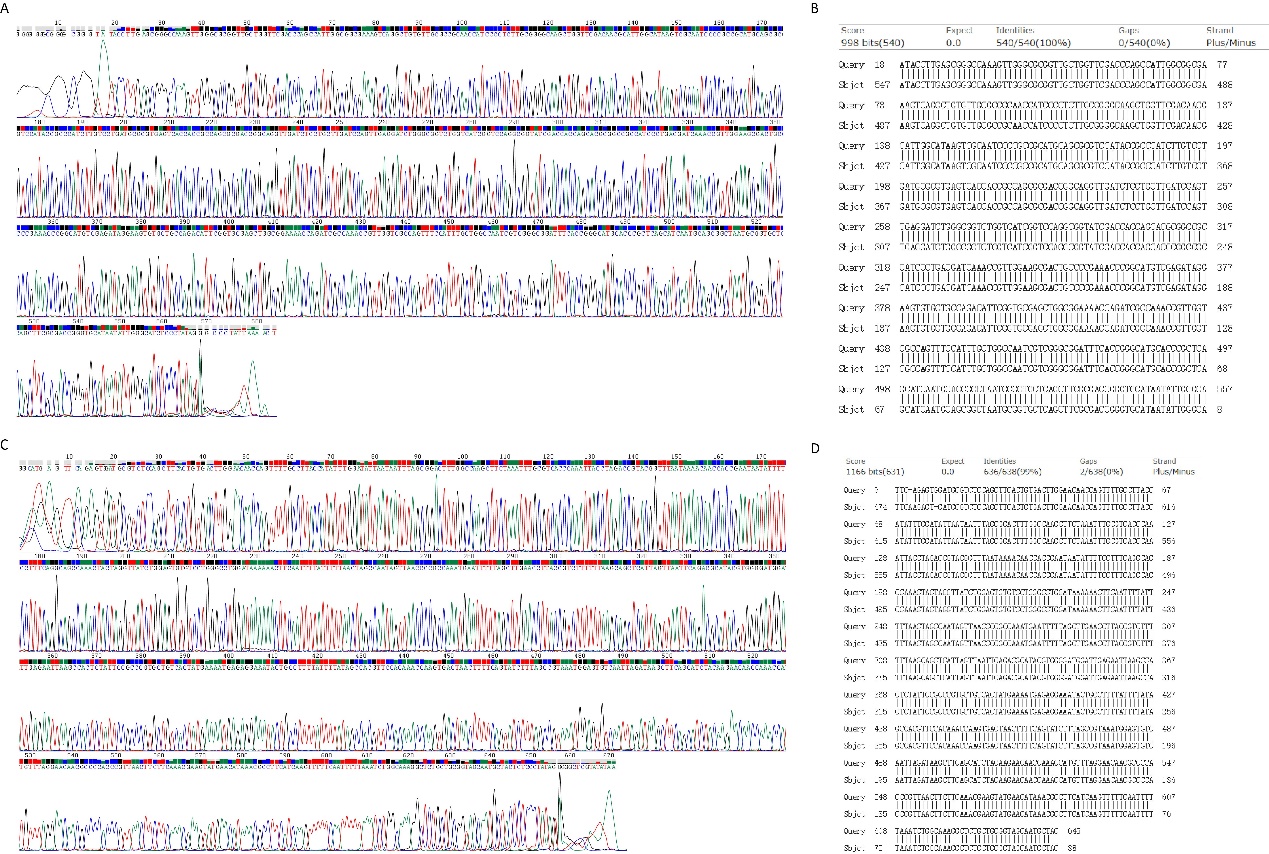
**

**Figure S2. Sequencing analysis of the fragments from S1-PFGE gel recovery PCR of AZS099.** The plasmid pAZS099-NDM-IMP separated by S1 nuclease treatment and PFGE was then recovered and used as the templates for PCR amplification and Sanger sequencing**.** (A) Sequence of *bla*_NDM-1_ from PCR amplifications. (B) Sequence alignment between *bla*_NDM-1_ fragment from PCR amplifications and *bla*_NDM-1_ gene in pAZS099-NDM-IMP. (C) Sequence of *bla*_IMP-4_ from PCR amplifications. (D) Sequence alignment between *bla*_IMP-4_ fragment from PCR amplifications and *bla*_IMP-4_ gene in pAZS099-NDM-IMP.

**Figure S3**

**
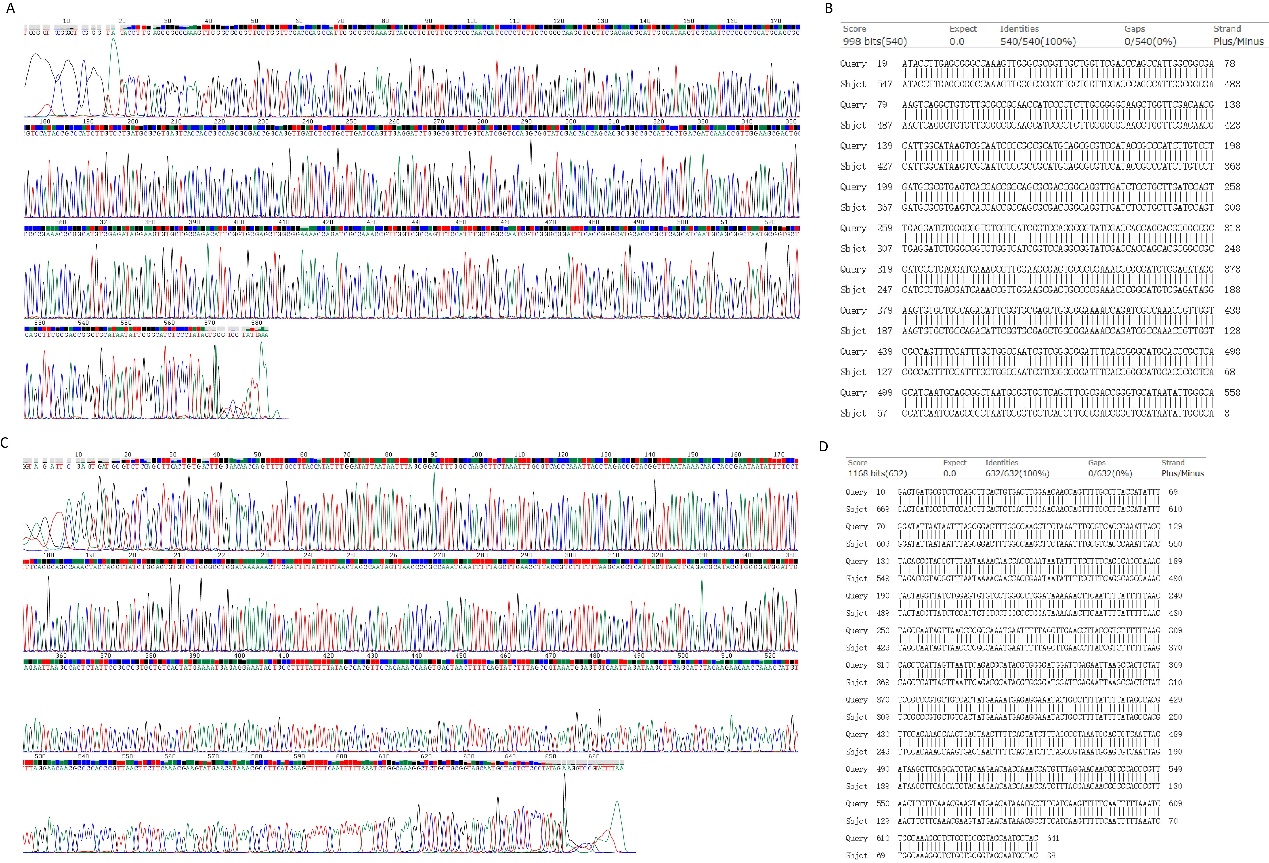
**

**Figure S3. Sequencing analysis of the fragments from S1-PFGE gel recovery PCR of the transconjugant EC600-pAZS099-NDM-IMP.** The plasmid pAZS099-NDM-IMP separated by S1 nuclease treatment and PFGE was then recovered and used as the templates for PCR amplification and Sanger sequencing**.** (A) Sequence of *bla*_NDM-1_ from PCR amplifications. (B) Sequence alignment between *bla*_NDM-1_ fragment from PCR amplifications and *bla*_NDM-1_ gene in pAZS099-NDM-IMP. (C) Sequence of *bla*_IMP-4_ from PCR amplifications. (D) Sequence alignment between *bla*_IMP-4_ fragment from PCR amplifications and *bla*_IMP-4_ gene in pAZS099-NDM-IMP.

**Figure S4**


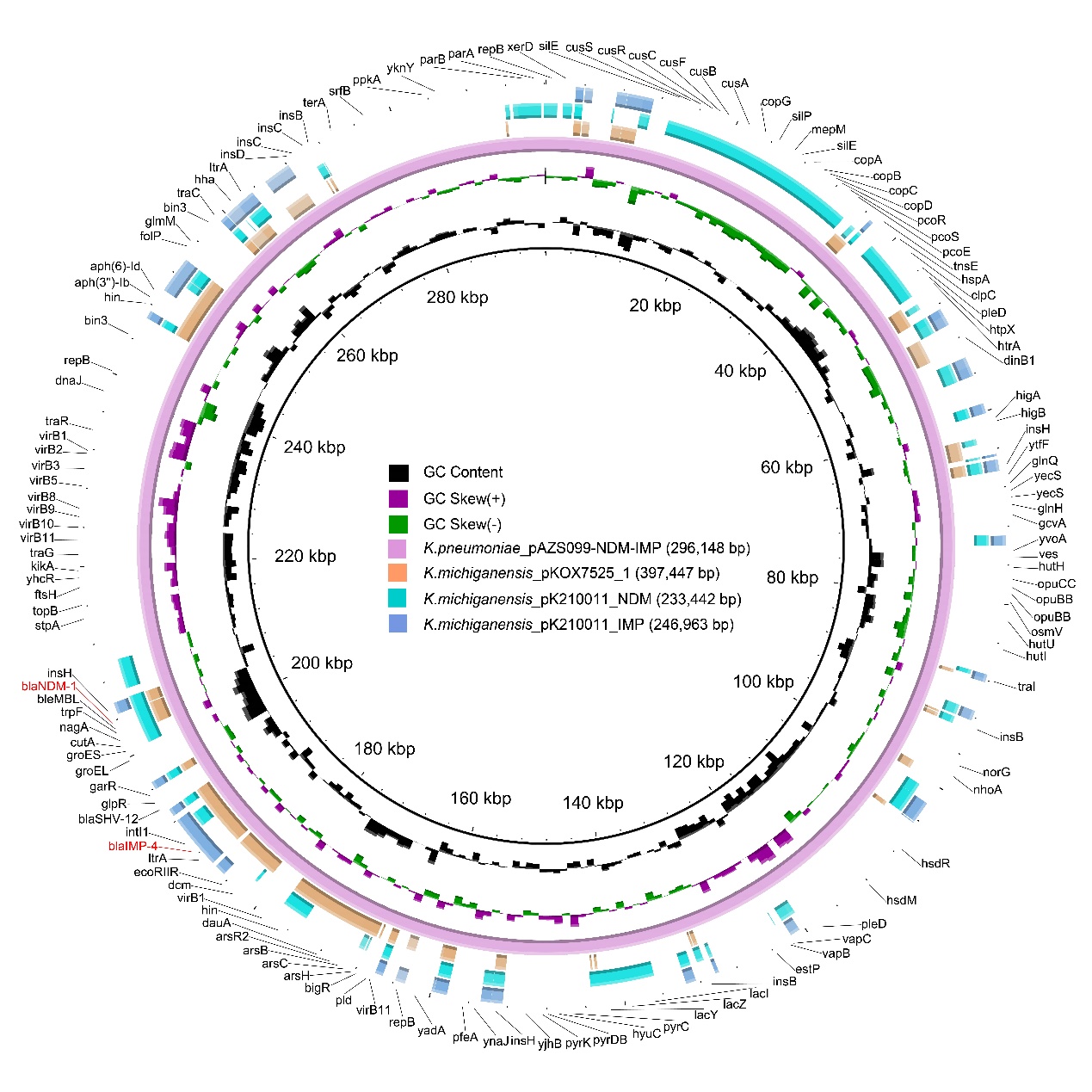


**Figure S4.** **Structural comparison of pAZS099-NDM-IMP with pKOX7525_1, pK210011_NDM, and pK210011_IMP.** Alignments of resemble plasmids are shown as concentric rings. The outermost shows the main coding genes of pAZS099-NDM-IMP. *bla*_NDM-1_ and *bla*_IMP-4_ are highlighted in red.

**Figure S5**


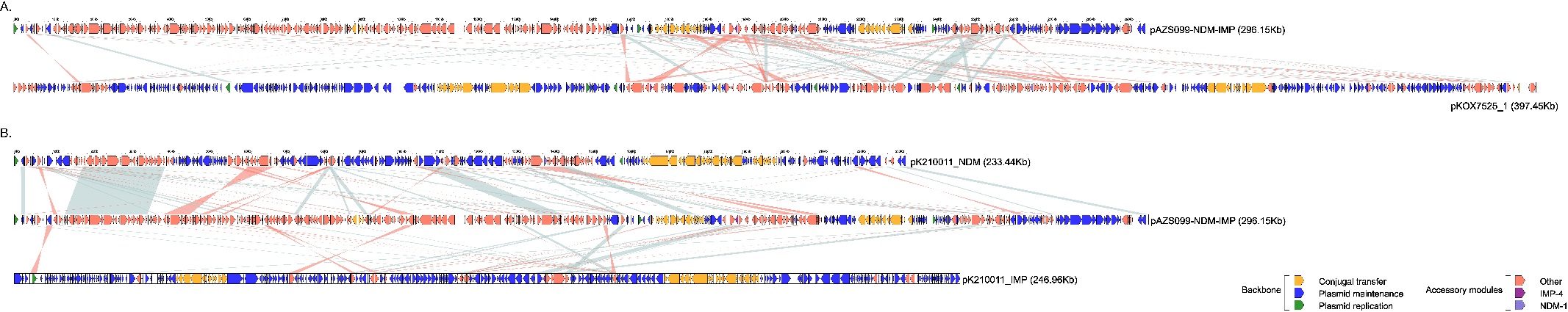


**Figure S5.** **Linear comparison of plasmid pAZS099-NDM-IMP with pKOX7525_1, pK210011_NDM, and pK210011_IMP.** (A) Linear comparison of plasmid pAZS099-NDM-IMP with pKOX7525_1 from *K. michiganensis* isolate KOX7525 [1]. (B) Linear comparison of plasmid pAZS099-NDM-IMP with pK210011_NDM and pK210011_IMP from *K. michiganensis* isolate K210011 [2]. Genes are denoted by arrows. Genes, mobile elements, and other features are colored based on functional classification. Shading denotes the regions with high homology (95% nucleotide identity).

**References**

[1] Li X, He J, Jiang Y, Peng M, Yu Y, Fu Y. Genetic Characterization and passage instability of a hybrid plasmid co-harboring *bla*_IMP-4_ and *bla*_NDM-1_ reveal the contribution of insertion sequences during plasmid formation and evolution. Microbiol Spectr (2021) 9: e0157721.

[2] Zhang Y, Gu D, Yang X, et al. Emergence and genomic characterization of a KPC-2-, NDM-1-, and IMP-4-producing Klebsiella michiganensis isolate. Front Microbiol (2022). 12:762509.
